# Effect of calcium ionophore (A23187) on embryo development and its safety in PGT cycles

**DOI:** 10.3389/fendo.2022.979248

**Published:** 2023-01-04

**Authors:** Junya Zhang, Guidong Yao, Tongwei Zhang, Jingyi Hu, Guang Yang, Jiahuan He, Qina He, Huiying Fan, Yucheng Bai, Yingpu Sun

**Affiliations:** ^1^ Center for Reproductive Medicine, The First Affiliated Hospital of Zhengzhou University, Zhengzhou, China; ^2^ Henan Key Laboratory of Reproduction and Genetics, The First Affiliated Hospital of Zhengzhou University, Zhengzhou, China

**Keywords:** calcium ionophore (A23187), fertilization failure, embryo development potential, chromosome aneuploidy, pregnancy outcome

## Abstract

**Background:**

Intracytoplasmic sperm injection (ICSI) has tremendous advantages for resolving the problem of male infertility. However, ICSI fertilization can fail in some patients because of various reasons, primarily because of the failure of oocyte activation. Oocytes have been activated using calcium ionophore (A23187) in previous clinical cases of ICSI fertilization failure. However, studies on the efficiency of calcium ionophore (A23187) activation, its effects on the developmental potential of embryos, and its effects on pregnancy outcomes after embryo transfer are relatively limited.

**Methods:**

In this study, we investigated the safety and long-term efficacy of calcium ionophore (A23187) by analyzing its effects on fertilization, embryonic development, aneuploidy, and pregnancy outcomes in patients undergoing preimplantation genetic testing (PGT) cycles.

**Results:**

Comparative analyses of the activation followed by PGT (A-PGT) and PGT groups revealed no significant differences between the oocyte cleavage rate and high-quality embryo rate (98.19% vs. 98.63% and 63.13% vs. 68.39%, respectively, *p* > 0.05). Although the blastocyst formation rate was significantly lower in the A-PGT group than that in the PGT group (52.22% vs. 59.90%, *p* < 0.05), no significant difference was observed in the blastocyst aneuploidy rates of the two groups (24.49% vs. 24.55%, *p* > 0.05). Furthermore, no significant differences were observed between the two groups in terms of the live birth rate (43.75% vs. 52.99%), week of delivery, and birth weight of the infants after transfer of euploid blastocysts (*p* > 0.05). Furthermore, the 2PN rate, oocyte cleavage rate, blastocyst formation rate, and live birth rate were found to be significantly lower in the A-ICSI group than those in the ICSI group (*p* < 0.01), but there was no significant difference between the two groups in the week of delivery and birth weight of live births (*p* > 0.05).

**Discussion:**

These results suggest that the use of calcium ionophore (A23187) activation as an option in cases of ICSI fertilization failure does not affect the ploidy of developing blastocysts and has no significant effects on the week of delivery or birth weight after transfer. Thus, we provide a scientific basis for the clinical safety of oocyte activation using calcium ionophore (A23187).

## 1 Introduction

Intracytoplasmic sperm injection (ICSI) is a fertilization technique wherein sperm is directly injected into the oocyte cytoplasm using a microinjector. ICSI fertilization bypasses the natural fertilization process of sperm crossing the zona pellucida, thereby changing the conventional paradigm of *in vitro* fertilization, and is effective for treating male infertility owing to male factors such as severe oligospermia, asthenospermia, teratospermia, and obstructive azoospermia. Since the first successful case of ICSI in 1992, ICSI has helped millions of families bear children (1) and has become a major tool for treating male infertility while overcoming the failures of conventional *in vitro* fertilization.

Despite the high fertilization and oocyte cleavage rates achieved in ICSI, 1–5% patients face the risk of total fertilization failure, with failure of oocyte activation being a major factor (2). Prior to fertilization, cyclin B and cyclin-dependent kinase 1 (CDK1) arrest oocytes in meiosis II (MII). Post-fertilization oocyte activation and resumption of meiosis depend on the maintenance of high levels of intracellular calcium ions within hours of fertilization, which is also known as calcium oscillations. Studies have shown that calcium oscillations are primarily initiated by sufficient amounts of phospholipase C zeta (PLCζ) released from the posterior region of the sperm acrosome (3). PLCζ hydrolyzes PIP2 into IP3 and DAG. IP3 then binds to receptors on the endoplasmic reticulum, causing calcium ions to flow from the calcium pool of the endoplasmic reticulum into the cytoplasm, leading to an increase in the intracellular calcium ion levels; this in turn stimulates calcium-dependent protein kinases, leading to a cascade response of cortical granule cytosolic and zona pellucida (4). DAG further stimulates and maintains these calcium oscillations through protein kinase C. Under normal conditions, the braking step during ICSI damages the plasma membrane of the sperm cell, leading to easier release of PLCζ, and thus, to calcium shock in the oocytes after ICSI fertilization. Nevertheless, oocyte activation fails in some patients. Some studies suggest that this may be due to the lack of PLCζ in sperm or mutations in the *PLCζ* gene (5). In addition, even sperm with normal PLCζ levels differ in their ability to activate oocytes (6).

To address the problem of oocyte fertilization failure or low fertilization rates, researchers have attempted to improve the clinical outcomes of patients with recurrent fertilization failure using artificial oocyte activation (AOA) techniques, including the use of the natural activator PLCζ, the artificial activator calcium ionophore (A23187), ionomycin, strontium chloride, and electrical pulse activation (7). Human recombinant PLCζ—a natural activator—can produce a calcium oscillation pattern in oocytes, which is similar to that produced during natural fertilization; however, the possibility that this may affect subsequent embryonic development cannot be ruled out, and therefore, it is not widely used in clinical practice. Electrical pulse activation is performed using a direct current, which causes the movement of charged proteins on the cell membrane, creating pores on the cell membrane surface that facilitate the entry of calcium ions from the culture medium into the cell, which in turn generates calcium oscillations to activate the oocyte (8). Electrical pulse activation requires special equipment and causes some degradation of oocytes; therefore, it is not widely used clinically. Sr^2+^, a divalent cation similar to Ca^2+^, has been used to achieve high rates of embryonic cleavage and blastocyst formation in parthenogenetic activation and somatic cell nuclear transfer in mouse experimental models (9); however, there are major differences in the activation efficiency of Sr^2+^ in human and mouse oocytes. In contrast, calcium ionophore (A23187) and ionomycin can activate oocytes by enhancing the permeability of the cell membrane to calcium ions. The first successful birth of a baby using ICSI combined with calcium ionophore (A23187) activation was reported in 1995 (10).

Previously, we used calcium ionophore (A23187) for rescue activation of unfertilized oocytes observed the day after ICSI and found that some percentage of high-quality blastocysts with normal karyotypes were obtained and healthy live-born offspring were obtained after embryo transfer. In addition, a time-lapse monitoring (TLM) system was used to analyze the timing of embryonic developmental; we found that rescue activation of unfertilized oocytes using calcium ionophore (A23187) did not affect the timing of development of the embryos after activation (11). These results suggest that the rescue activation of unfertilized oocytes using calcium ionophore (A23187) after ICSI may enable some patients to obtain usable embryos in the first ICSI cycle, reducing the financial burden and waiting time for patients, thus providing a basis for the widespread use of calcium ionophore (A23187) rescue activation for assisted reproduction.

Calcium ionophore (A23187) is a highly selective calcium ion carrier that is frequently used in the AOA process. A meta-analysis showed that calcium ionophore (A23187) helps promote oocyte activation and improves the rates of fertilization, blastocyst formation, embryo implantation, and live births (12). Miller et al. (2016) analyzed and compared the effects of ICSI alone and ICSI combined with calcium ionophore (A23187) in singleton and twin births and found no differences in birth defects between the two groups (13). Another study focused on the effects of calcium ionophore (A23187) activation on the probability of errors in meiosis II in oocytes and found no significant difference from the group treated with ICSI alone without activation (14). Nevertheless, there is no study on the effect of calcium ionophore (A23187) activation on embryonic aneuploidy in human PGT cycle; thus, the possible subsequent gene expression and epigenetic changes caused by calcium ionophore (A23187) activation must be investigated in detail.

The safety of calcium ionophore (A23187), the differences between calcium ion release induced during AOA, and the physiological calcium ion level, which is a key component for initiating downstream events during oocyte activation, are still under debate. Differences in the elevation of calcium ion concentrations and the magnitude and frequency of this elevation may induce differences in gene expression and even epigenetic defects, thus warranting attention (15, 16). Therefore, further systematic studies are necessary to investigate the safety of the clinical use of calcium ionophore (A23187) in terms of the AOA activation efficiency, embryonic developmental potential, chromosomal status, and effects on offspring.

In this study, we investigated the effects of calcium ionophore (A23187) on oocyte fertilization, embryonic developmental potential, aneuploidy, and post-transfer pregnancy outcomes by including patients undergoing ICSI and preimplantation genetic testing (PGT) cycles and then analyzed the effects of calcium ionophore (A23187) oocyte activation on embryonic development and the long-term safety to provide a basis for the clinical application of calcium ionophore (A23187) for oocyte activation.

## 2 Materials and methods

### 2.1 Sample collection

In the study, the data of 506 patients treated with assisted reproductive technology at the Reproductive Medicine Center of the First Affiliated Hospital of Zhengzhou University were retrospectively analyzed. The inclusion criteria were as follows: age 22–38 years and BMI 16–28 kg/m^2^ for both partners. The exclusion criteria were as follows: (1) specific ovarian pathology, including polycystic ovary syndrome and premature ovarian failure; (2) disorders such as endometriosis, structural abnormalities of the uterus (e.g., unicornuate uterus and double uterus), and cervical insufficiency; and (3) patients with pelvic tuberculosis and hydrocele in the fallopian tubes. Included cases were divided into four groups based on the purpose of the experimental study: group treated with ICSI alone (ICSI group), group treated with calcium ionophore (A23187) activation following ICSI (A-ICSI group), group with blastocysts that originated from patients undergoing ICSI and preimplantation genetic testing alone (PGT group), and group with blastocysts that originated from patients undergoing ICSI, treated with calcium ionophore (A23187) activation, and followed by preimplantation genetic testing (A-PGT group). Among them, 234 patients in the ICSI group underwent ICSI for male factors alone, 76 patients in the A-ICSI group underwent activation with calcium ionophore (A23187) directly after ICSI in the current cycle owing to failed ICSI or sperm malformation, and 172 patients in the PGT group underwent preimplantation genetic testing of blastocysts derived from ICSI. Furthermore, 172 patients in the PGT group underwent PGT of blastocysts derived from ICSI, and 24 patients in the A-PGT group underwent calcium ionophore (A23187) activation after ICSI and underwent pre-implantation genetic testing because of failed ICSI or sperm malformation in the previous cycle.

### 2.2 Intracytoplasmic sperm injection (ICSI)

Thirty-six hours after the patients were injected with recombinant hCG (250 μg) (Serono. Ltd., Switzerland), transvaginal B-mode ultrasound guided puncture was performed to harvest cumulus oocyte complexes (COCs). The harvested COCs were immediately added to G-IVF Plus (Vitrolife, Goteborg, Sweden) culture medium for 1–2 h in an incubator set at 37°C and 6% CO_2_.

Semen was obtained by masturbation, and Spermgrad (Vitrolife) density gradient centrifugation (90% and 45%) and G-IVF Plus centrifuge wash were used to collect sperm from liquefied semen. Fine-needle aspiration of the epididymides or testes was performed to collect samples from obstructive azoospermia patients. Sperm was collected through epididymis sperm density gradient centrifugation and G-IVF Plus centrifuge wash. Testicular tissues obtained from testicular fine-needle aspiration were homogenized using a 1 mL needle in G-IVF Plus solution, and sperm was collected after direct centrifugation based on a previous study (11).

COCs cultured *in vitro* for 1–2 h were digested using a fine needle with a diameter of 150 μm (Sunlight, FL, USA), and hyaluronidase (Vitrolife) was used to remove the cumulus cell around the COCs. Mature oocytes obtained after cumulus cell removal were added to G-IVF Plus, and culturing was continued for 1–2 h before ICSI fertilization. After ICSI, the fertilized oocytes were cultured in G-1 Plus (Vitrolife) culture droplets covered with parafilm oil (Vitrolife). Culturing was performed in an incubator set at 37°C and 6% CO_2_.

### 2.3 Artificial oocyte activation

Oocytes that were fertilized after ICSI, which required activation, were transferred to the G-1 Plus culture medium containing 10 μM calcium ionophore (A23187) (Sigma, MO, USA) prepared in advance and incubated for 10 min. The activated oocytes were washed with G-1 Plus thrice before transferring them into G-1 Plus culture droplets to continue culture.

### 2.4 Pronuclear observation and embryo culture

Sixteen to eighteen hours (Day 1, D1) after ICSI fertilization, the oocyte fertilization status was observed under an inverted microscope (TE2000-U, Nikon, Japan). Normal fertilized oocytes presented two distinct pronuclei (2PN) under an inverted microscope. 2PN rate (%) = number of 2PN zygotes/number of MII oocytes × 100%. Then, 44–46 h (Day 2, D2) after ICSI fertilization, the cleavage of normally fertilized embryos was observed, and 68–70 h (Day 3, D3) after ICSI fertilization, the developmental status of embryos was observed. The cleavage stage embryos were scored based on Peter’s scoring system (17), and grade I and II embryos were considered to be good-quality embryos. D2 cleavage rate (%) = number of normally fertilized cleavage stage embryos/number of 2PN zygotes × 100%; D3 high-quality embryo rate (%) = number of D3 high-quality embryos/number of 2PN zygotes × 100%.

For embryos that continued to the blastocyst stage, embryos that developed to D3 were transferred to G-2 Plus (Vitrolife) culture droplets, covered with paraffin oil, and cultured to Day 5 (D5) or Day 6 (D6). The formed blastocysts were graded according to the Gardner blastocyst scoring system (18). The blastocyst quality grade was generally 3BB and above for D5 embryos and 4BB and above for D6 embryos. Blastocyst formation rate (%) = Total number of formed blastocysts/Number of cultured embryos ×100%, Blastocyst formation rate from high-quality embryos (%) = Number of formed blastocysts from high-quality embryos/Number of cultured high-quality embryos ×100%, Blastocyst formation rate from non-high-quality embryos (%) = Number of formed blastocysts from non-high-quality embryos/Number of cultured non-high-quality embryos ×100%.

### 2.5 Biopsy of trophectoderm cells and chromosome analysis

In the afternoon of Day 4 of embryo development, a laser-assisted hatching system (ZILOS-tk, Hamilton Thorne Biosciences, Beverly, MA, USA) was used to make a hole in the zona pellucida far from the inner cell mass (ICM) to promote the outgrowth of trophoblast cells (TE). On D5 or D6, blastocysts showed significant expansion and protrusion of the blastocoel. Under an inverted microscope, a 25 μm diameter biopsy needle (Origio, VA, USA) was used to extract 3–5 TEs for subsequent chromosome aneuploidy analysis. NGS was used to detect chromosome aneuploidy in the extracted cells. Details of the method used have been reported in our previous study (19).

### 2.6 Clinical follow up

Serum β-hCG levels were measured 14 d after embryo transfer, and the subject was considered to have biochemical pregnancy if β-hCG ≥ 50 U/L. Ultrasound examinations were performed 35 d after transfer, and clinical pregnancy was confirmed if primitive heart tube pulsation and gestational sac were present. Early miscarriage was diagnosed if the embryo development stopped or the expulsion of the products of conception occurred within 12 weeks of pregnancy. Live birth was diagnosed when the pregnancy continued for 28 weeks, and there was at least 1 surviving neonate. β-hCG positivity rate (%) = number of biochemical pregnancies/number of embryo transfer cycles ×100%; clinical pregnancy rate (%) = number of clinical pregnancies/number of embryo transfer cycles ×100%; embryo transfer rate (%) = number of gestational sacs/number of embryos transferred ×100%; live birth rate (%) = number of live births/number of embryo transfer cycles × 100%. The delivery status and birth weight of the fetus were also recorded.

### 2.7 Statistical analysis

SPSS 22.0 was used for the statistical analysis of data regarding oocyte maturation rate, fertilization rate, and cleavage rate of each group. GraphPad Prism 9.0 software was used for data visualization. Quantitative data were expressed as the mean ± standard deviation (x^–^ ± sd); student’s t-test was performed for comparisons between two groups of data; one-way ANOVA was used for comparing the differences between multiple groups; and the least significant difference (LSD) and Tamhane methods were used for post-hoc comparative analysis after ANOVA. Rates were compared using columnar analysis with chi-square or Fisher’s exact test. Differences with *p* < 0.05 were considered statistically significant.

## 3 Results

### 3.1 General characteristics of the patients

A total of 506 patients were included in this study, including 234 in the ICSI group, 76 in the A-ICSI group, 172 in the PGT group, and 24 in the A-PGT group. A comparison of the general clinical characteristics of the patients revealed that the differences between the groups in terms of age, anti-Müllerian hormone (AMH), basal FSH, basal estradiol (E_2_), testosterone (T), serum free triiodothyronine (FT3), serum free thyroxine (FT4), and thyroid stimulating hormone (TSH) in the female patients were not statistically significant (*p* > 0.05) (**Table 1**). The BMI was significantly higher in the ICSI group than that in the PGT group (*p* < 0.05); the LH levels were significantly higher in the ICSI group than those in the PGT group (*p* < 0.05); the P levels were significantly higher in the PGT group than those in the ICSI group (*p* < 0.05); and the PRL levels were significantly lower in the PGT group than those in the A-ICSI group (*p* < 0.05).

**Table 1:** Comparison of the general clinical characteristics of patients in the four groups.

Groups	ICSI	A-ICSI	PGT	A-PGT
No. Age (year)	234 30.13 ± 2.61	76 29.26 ± 3.78	172 30.31 ± 2.62	24 28.75 ± 4.35
BMI (kg/m^2^)	22.78 ± 1.68	23.52 ± 3.06^*^	22.52 ± 1.62	23.02 ± 3.83
AMH (ng/ml)	4.07 ± 2.75	3.62 ± 2.63	4 ± 2.47	5.2 ± 3.54
Basal FSH (mIU/ml)	6.16 ± 1.81	6.65 ± 2.01	6.3 ± 1.92	6.89 ± 1.33
Basal LH (mIU/ml)	5.35 ± 3.52^*^	4.9 ± 3.13	4.05 ± 3.15	5.19 ± 2.83
Basal E_2_ (pg/ml)	38.66 ± 16.84	34.41 ± 14.16	37.46 ± 15.09	40.59 ± 18.52
Basal P (ng/ml)	0.39 ± 1.25	0.41 ± 0.32	0.72 ± 1.16^*^	0.42 ± 0.32
Basal PRL (ng/ml)	17.2 ± 7.2	18.31 ± 7.39	14.94 ± 5.73^*^	16.21 ± 4.91
Basal T (ng/ml)	0.27 ± 0.14	0.29 ± 0.16	0.3 ± 0.16	0.35 ± 0.23
FT3 (pmol/ml)	5.25 ± 0.65	5.21 ± 0.66	5.15 ± 0.64	5.27 ± 0.76
FT4 (pmol/ml)	11.46 ± 1.67	11.38 ± 1.55	11.78 ± 2.16	12.43 ± 2.74
TSH (uIU/ml)	2.46 ± 1.2	2.47 ± 1.13	2.28 ± 1.09	3 ± 1.81

Data are expressed as the means ± standard deviation. BMI, body mass index; AMH, anti-Müllerian hormone; FSH, follicle-stimulating hormone; LH, luteinizing hormone; E_2_, estradiol; P, progesterone; PRL, prolactin; T, testosterone; FT3, serum free triiodothyronine; FT4, serum free thyroxine; TSH, thyroid stimulating hormone. ^*^p < 0.05.

### 3.2 Effects of calcium ionophore (A23187) on embryonic development in patients undergoing preimplantation genetic testing

To investigate the effect of calcium ionophore (A23187) on embryonic development in patients undergoing PGT cycles, we analyzed 2548 MII oocytes from 172 patients in the PGT-only group and 390 MII oocytes from 24 patients in the A-PGT group with calcium ionophore (A23187) activation and compared them for embryonic development after fertilization. No significant differences were observed between the two groups in terms of age, BMI, FSH, and other general clinical characteristics (*p* > 0.05) (**Supplementary Table 1**).

A comparative analysis of fertilization, oocyte cleavage, and blastocyst formation between the PGT and A-PGT groups showed that the normal fertilization rate was significantly lower in the A-PGT group than that in the PGT group (56.67% vs. 77.47%, *p* = 0.000); however, there was no significant difference in the D2 oocyte cleavage rate and D3 high-quality embryo rate between the two groups (*p* > 0.05) (**Table 2**). Further comparisons of the embryonic developmental potential of the A-PGT and PGT groups revealed that the blastocyst formation rate was significantly higher in the PGT group than that in the A-PGT group (59.90% vs. 52.22%, *p* < 0.05), and the blastocyst formation rate after the blastocyst culture of high-quality embryos in the PGT group was significantly higher than that in the A-PGT group (70.32% vs. 54.01%, *p* = 0.000). Interestingly, the blastocyst formation rate after blastocyst culture was significantly lower in the PGT group than that in the A-PGT group (29.76% vs. 48.48%, *p* < 0.01).

**Table 2:** Effects of A23187 activation on oocyte fertilization and embryonic development in patients undergoing PGT.

Groups	PGT	A-PGT		P value	
Mean oocytes retrieved	17.56 ± 0.56	19.38 ± 1.56		0.259	
MII rate (%)	84.37% (2548/3020)	83.87% (390/465)		0.783	
2PN rate (%)	77.47% (1974/2548)	56.67% (221/390)		0.000	
D2 cleavage rate (%)	98.63% (1947/1974)	98.19% (217/221)		0.820	
D3 high-quality embryo rate (%)	69.39% (1351/1947)	63.13% (137/217)		0.059	
Blastocyst formation rate (%)	59.90% (1089/1818)	52.22% (106/203)		0.035	
Blastocyst formation rate from high-quality embryos (%)	70.32% (950/1351)	54.01% (74/137)		0.000	
Blastocyst formation rate from non-high-quality embryos (%)	29.76% (139/467)	48.48% (32/66)		0.002	

Mean oocytes retrieved indicates the total number of oocytes retrieved from all patients in this group/number of patients. MII rate (%) = total number of mature oocytes from all patients in this group/total number of all oocytes × 100%. D2, day 2. D3, day 3. p < 0.05 indicates a statistically significant difference between the groups.

### 3.3 Effects of calcium ionophore (A23187) activation on the chromosomal status of blastocysts derived from PGT patients

**Figure 1:**
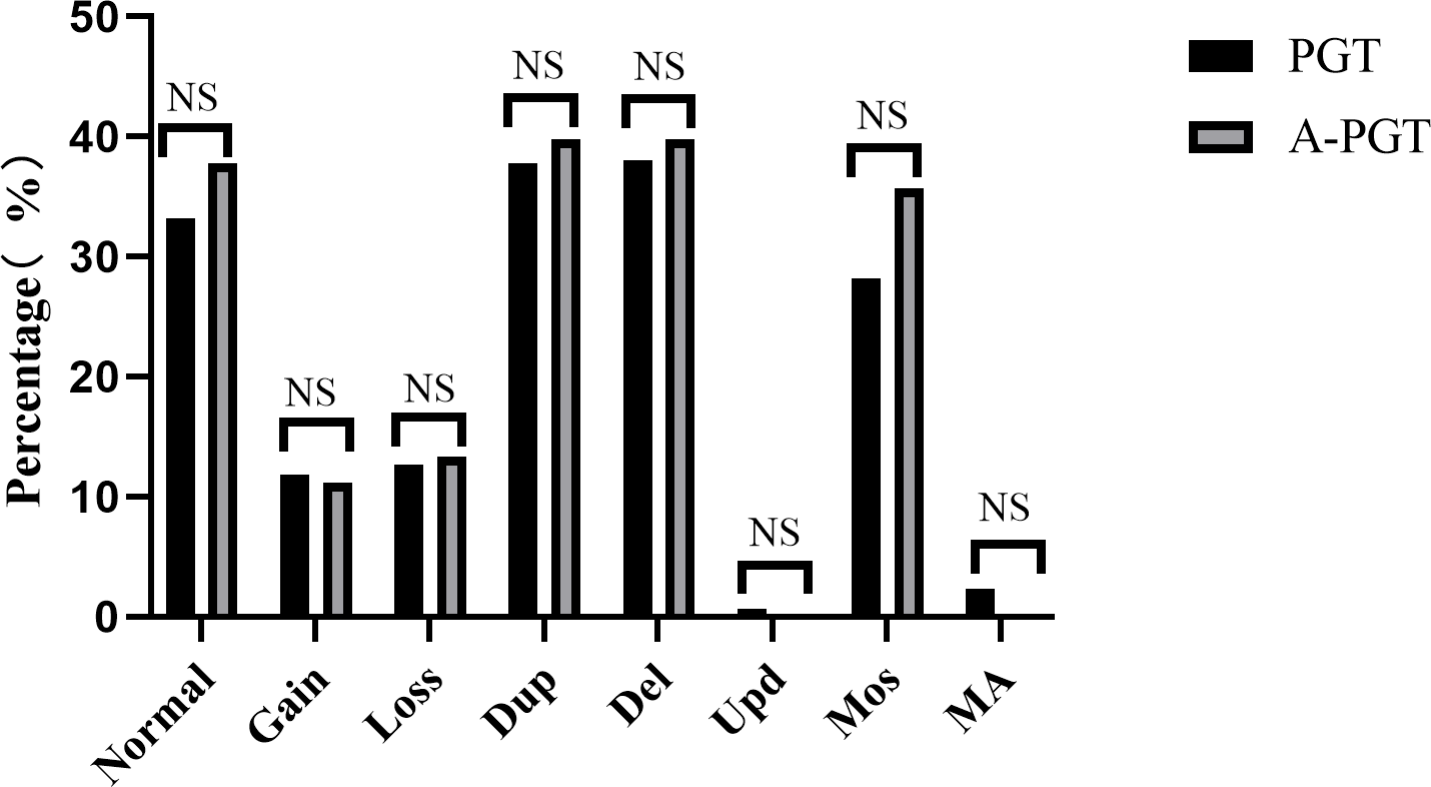
Comparative analysis of the chromosomal status of blastocysts in patients between the PGT and A-PGT group. Bars in the graph indicate percentages. Normal karyotype: 46, XX or 46, XY. Gain, increased copy number of the entire chromosome. Loss, at least one entire chromosome is missing. Dup, duplication of a chromosomal fragment. Del, loss of a chromosomal fragment. Upd, uniparental diploidy. Mos, mosaicism. MA, Multiple chromosomal abnormalities containing both duplication or deletion of chromosomal segments and mosaicism. NS indicates no statistically significant difference between the two groups.

Therefore, we analyzed and compared the karyotype results from trophectoderm cell biopsy of 1051 blastocysts in the PGT group and 98 blastocysts in the A-PGT group. No significant differences were observed in the proportion of euploidy between the blastocysts in the PGT and A-PGT groups (33.2% vs. 37.8%, *p* > 0.05) (**Figure 1**). Further analysis of aneuploid blastocysts revealed no significant differences between the PGT and A-PGT groups in terms of chromosome number, duplication or deletion of chromosome segments, uniparental diploidy, and incidence of mosaicism (*p* > 0.05). Interestingly, chromosomal abnormalities in both groups primarily manifested as deletions and duplications of chromosomal segments, at 75.74% and 79.59%, respectively.

To investigate the safety of calcium ionophore (A23187) oocyte activation, we performed a comparative analysis of the clinical pregnancies and births after thawing and transferring 137 blastocysts from the PGT group and 20 blastocysts from the A-PGT group that were confirmed to be chromosomally normal from pre-implantation genetic diagnosis. The results indicated no statistically significant difference between the clinical pregnancy rates in the PGT and A-PGT groups (63.50% vs. 50.00%, *p* > 0.05) (**Table 3**). The cases that had reached delivery dates, including 117 and 16 cases in the PGT and A-PGT groups, respectively, as well as 62 and 7 live births in the two groups, respectively, were followed up. There was no significant difference between the two groups in terms of the live birth rate (52.99% vs. 43.75%, *p* > 0.05). Further, follow-up of the neonates showed no significant differences (*p* > 0.05) between the two groups in terms of gestational age at birth (38.74 ± 1.46 weeks vs. 39.00 ± 1.25 weeks) and birth weight (3348.52 g ± 496.61 g vs. 3414.29 g ± 430.81 g) (**Table 3**).

**Table 3:** Comparison of the pregnancy outcomes and birth week between PGT and A-PGT groups.

Groups	PGT	A-PGT	P value
Blastocysts transferred (n)	137	20	–
β-hCG positive rate (%)	70.8% (97/137)	65.00% (13/20)	0.597
Clinical pregnancy rate (%)	63.50% (87/137)	50.00% (10/20)	0.246
Live birth rate (%)	52.99% (62/117)	43.75% (7/16)	0.488
Birth (wk)	38.74 ± 1.46	39.00 ± 1.25	0.659
Weight (g)	3348.52 ± 496.61	3414.29 ± 430.81	0.738

Only one chromosomally normal blastocyst was transferred in each PGT patient at our center, so the number of blastocysts transferred, number of transfer cycles, clinical pregnancy rate, and rate of implantation were the same. Values are expressed as the mean ± SD, and p > 0.05 indicates no statistically significant difference.

### 3.4 Comparison of early embryonic development and pregnancy outcomes between A-ICSI and A-PGT groups

To investigate whether the effects of artificial oocyte activation using calcium ionophore (A23187) on embryonic development differed between patients with ICSI and those undergoing PGT, a further comparative analysis was performed for the 76 A-ICSI cycles and 24 A-PGT cycles included in this study. No significant differences were observed in the clinical characteristics, such as age, BMI, and basal FSH between the two groups (*p* > 0.05), except for the AMH level in the A-PGT group, which was significantly higher than that in the A-ICSI group (*p* < 0.05) (**Supplementary Table 2**).

An analysis of embryonic development, including fertilization rate and cleavage rate in the A-ICSI and A-PGT groups, showed that the mean number of oocytes obtained and the MII ratio were significantly higher in the A-PGT group than those in the A-ICSI group (*p* < 0.05); however, no statistically significant difference was observed between the two groups in terms of the 2PN rate, D2 cleavage rate, and D3 high-quality embryo rate (*p* > 0.05) (**Table 4**). Interestingly, the blastocyst formation rate was significantly higher in the A-PGT group than that in the A-ICSI group (52.22% vs. 43.08, *p* < 0.05).

**Table 4:** Comparison of embryo development between A-ICSI and A-PGT groups.

Groups	A-ICSI	A-PGT	P value
Mean oocytes retrieved	14.83 ± 7.26	19.38 ± 7.63	0.010^*^
MII rate (%)	78.35% (883/1127)	83.87%(390/465)	0.012^*^
2PN rate (%)	59.68% (527/883)	56.67% (221/390)	0.314
D2 cleavage rate (%)	96.96% (511/527)	98.19% (217/221)	0.484
D3 high-quality embryo rate (%)	56.56% (289/511)	63.13% (137/217)	0.099
Blastocyst formation rate (%)	43.08% (137/318)	52.22% (106/203)	0.042^*^

D2, day 2. D3, day 3. ^*^p < 0.05 indicates statistically significant differences.

### 3.5 Effects of calcium ionophore (A23187) activation on early embryonic development and pregnancy outcome in ICSI cycles

To investigate the effect of calcium ionophore (A23187) activation on embryo development after ICSI-only fertilization, the general clinical characteristics of patients included in the ICSI and A-ICSI groups were compared. The results showed that no statistically significant differences existed in the clinical characteristics between the two groups, except for BMI and basal FSH (*p* < 0.05) (**Supplementary Table 3**).

Further analysis of the fertilization and embryonic development potential of ICSI and A-ICSI groups showed that the 2PN rate, D2 cleavage rate, and blastocyst formation rate were significantly higher in the ICSI-only group than those in the A-ICSI group (*p* < 0.05); however, the D3 high-quality embryo rate did not differ significantly between the two groups (*p* > 0.05) (**Table 5**).

**Table 5:** Effects of A23187 activation on embryonic development after ICSI cycles.

Groups	ICSI	A-ICSI	P value
Mean oocytes retrieved	14.66 ± 6.18	14.83 ± 7.26	0.845
MII rate (%)	77.85% (2671/3431)	78.35% (883/1127)	0.725
2PN rate (%)	73.53% (1964/2671)	59.68% (527/883)	0.000^*^
D2 cleavage rate (%)	98.73% (1939/1964)	96.96% (511/527)	0.005^*^
D3 high-quality embryo rate (%)	61.22% (1187/1939)	56.56% (289/511)	0.055
Blastocyst formation rate (%)	52.38% (616/1176)	43.08% (137/318)	0.003^*^

D2, Day 2. D3, Day 3. ^*^p < 0.05 indicates statistical significance.

To investigate whether the effect of calcium ionophore (A23187) activation on pregnancy outcomes differed between the ICSI and A-ICSI groups, post-transfer follow-up analysis was performed on 287 embryos transferred from 182 patients in the ICSI group and 76 embryos transferred from 45 patients in the A-ICSI group. The results revealed that the post-transfer rates of fertilization (30.26% vs. 51.22%) and live births (26.32% vs. 43.90%) were significantly lower in the A-ICSI group than those in the ICSI-only group (*p* < 0.01) (**Table 6**). In contrast, the analysis of the gestational age at birth and weight of successfully delivered offspring in both groups did not present significant differences (*p* > 0.05). Furthermore, because of the differences in the number of embryos transferred between the two groups, we found no significant differences in the gestational age and weight at birth between the two groups for both singleton and twin births (*p* > 0.05) (**Supplementary Table 4**).

**Table 6:** Effect of A23187 activation on pregnancy outcome and offspring after an ICSI cycle.

Groups	ICSI	A-ICSI	P value
Patients transferred (n)	182	45	–
Implantation rate (%)	51.22% (147/287)	30.26% (23/76)	0.001
Live birth rate (%)	43.90% (126/287)	26.32% (20/76)	0.005
Birth (wk)	38.37 ± 1.93	37.96 ± 2.37	0.467
Weight (g)	3023.47 ± 649.98	2808.00 ± 742.07	0.179

Values are presented as mean ± SD. p > 0.05 indicates no statistical significance.

## 4 Discussion

Studies have shown that failure of oocyte activation is the principal cause of recurrent fertilization failure in ICSI (20), and calcium shock plays a major factor in successfully inducing oocyte activation (21). Sperm induce Ca^2+^ signaling in the oocyte cytoplasm, and the generation of Ca^2+^ signals trigger a subsequent series of physiological changes, such as the rapid depolarization of the oocyte oocyte membrane potential, release of cortical granules, chromosomal segregation, extrusion of the second polar body, decreased activity of kinases such as MPF/MAPK, translation of egg mRNA, pronucleus formation, and DNA replication. The orderly occurrence of these molecular events is also a hallmark of successful oocyte activation (22). Thus, the induction of an elevated intracellular Ca^2+^ concentration in oocytes through AOA is an alternative treatment for patients with recurrent fertilization failure in ICSI and can result in normal embryos.

Calcium ionophore (A23187) is widely used in clinical practice for AOA because of its ease of application and availability. Calcium ionophore (A23187) primarily increases the permeability of the cell membrane to allow the inward flow of extracellular Ca^2+^ into the cell, resulting in an increase in the Ca^2+^ concentration in the oocyte and thus oocyte activation (23). In addition, calcium ionophore (A23187) induces Ca^2+^ release from the Ca^2+^ pool in the endoplasmic reticulum of the oocyte in a manner similar to the IP3-mediated Ca^2+^ elevation induced by sperm. Most current studies suggest that calcium ionophore (A23187) can effectively improve the prognosis of patients with sperm abnormalities, increasing the rates of fertilization, implantation, and pregnancy (12, 24). However, some studies have found no significant improvement in the fertilization rates with calcium ionophore (A23187) activation after ICSI (25). The primary reason for this discrepancy is that different populations were included in different studies, and the reasons for their oocyte fertilization failure may not be identical. In addition, the relatively small sample size included in the studies may have affected the results.

Embryologists have been focusing on the safety of calcium ionophore (A23187) activation, particularly because the amplitude and frequency of calcium ionophore-induced calcium oscillations differ based on physiological conditions (26) and calcium oscillation has an important effect on chromosome segregation and other changes in genetic material (4). Capalbo et al. analyzed the effects of calcium ionophore (A23187) activation on MII oocytes and found that the incidence of aneuploidy after activation was 40.7%, consistent with the ratio observed for a group of women with the same age in related studies (14). That study indicated that calcium ionophore (A23187) activation did not have any subsequent effects on chromosome segregation. However, the number of patients included in that study was small, including 12 patients who donated 56 oocytes, and only activated oocytes were studied, chromosomal changes in the subsequent blastocyst formation stage in culture were not investigated. In this study, all 465 oocytes obtained from patients in A-PGT group were fertilized and tnen embryos were cultured to D5 or D6 for chromosome detection.In this study, we systematically analyzed the potential effects of calcium ionophore (A23187) on embryo development in patients undergoing PGT cycles and simultaneously performed a comparative analysis of chromosome aneuploidy in blastocysts, post-transfer embryo implantation, pregnancy outcomes, and birth status of neonates. Our findings showed that there was no significant difference in the ratio of normal chromosomes in blastocysts from the PGT and A-PGT groups (33.2% vs. 37.8%, *p* > 0.05), and most chromosomal abnormalities in the two groups involved the loss and duplication of chromosomal segments. However, there was no significant difference in the loss and duplication of chromosomal segments (75.74% vs. 79.59%, *p* > 0.05). Our results similarly showed that calcium ionophore (A23187) activation did not affect the chromosomes of blastocysts.

The embryo quality and developmental potential of embryos are also important aspects for evaluating the safety of calcium ionophore (A23187). In this study, no significant differences were observed in the rate of D3 good-quality embryos between the A-PGT and PGT groups and between the A-ICSI and ICSI groups; these results are consistent with those of a prospective randomized sibling oocyte study (25), showing that calcium ionophore (A23187) activation does not affect early embryonic development. We found that the blastocyst formation rate in the ICSI group was significantly higher than that in the A-ICSI group and the blastocyst formation rate of the PGT group was also significantly higher than that of the A-PGT group. This may be because of the abnormalities in the oocytes or sperms, which affected the developmental potential of the embryo. Ebner et al. compared the embryonic developmental outcomes of patients who underwent AOA with those of patients who did not. Results showed that AOA significantly increased the blastocyst formation rate (27). This result supports our hypothesis that calcium ionophore (A23187) activation does not have adverse effects on embryonic development. Interestingly, the good-quality embryo blastocyst formation rate in the A-PGT group was significantly lower than that in the PGT group and the non-good-quality embryo blastocyst formation rate in the A-PGT group was significantly higher than that in the PGT group. This suggests that calcium ionophore (A23187) activation can help increase the embryonic development potential of non-good-quality embryos.

We found that the blastocyst formation rate of the A-PGT group was significantly higher than that of the A-ICSI group. Furthermore, a significant difference was observed in the AMH levels between the two groups, and the mean number of oocytes harvested and the oocyte maturation rate of the A-PGT group with higher AMH levels were significantly higher than those of the A-ICSI group. The AMH expression level is related to the ovarian reserve size and is associated with ovarian functions and oocyte quality (28, 29). No abnormality in fertility was observed in most couples undergoing PGT, whereas abnormalities in fertility, such as in terms of sperm quality, were present in couples undergoing ICSI. This may be the reason for the differences in the blastocyst formation rate.

Calcium ionophore (A23187) is used for AOA, and the examination of whether calcium ionophore (A23187) affects post-transfer pregnancy outcomes and the health of successfully delivered infants are safety concerns for the clinical use of calcium ionophore (A23187). A prospective multicenter study compared the pregnancy rate and live birth rate of patients who underwent calcium ionophore (A23187) activation with those in the preceding cycle without calcium ionophore (A23187) activation. The results showed that the calcium ionophore (A23187) activation group had lower embryo transfer cancellation rate (27). A meta-analysis of 14 studies and 1521 IMSI or ICSI cycles showed that calcium ionophores significantly increased the overall pregnancy rate of couples with low fertilization rates (12).

We have showed that there are no significant differences in the clinical pregnancy rate (50.00% vs. 63.50%) and live birth rate (43.75% vs. 52.99%) between PGT patients in the calcium ionophore (A23187) activation group and non-activation group (*p* > 0.05). However, the implantation rate and live birth rate in the A-ICSI group were significantly lower than those in the ICSI group (implantation rate: 30.26% vs. 51.22%, *p* < 0.01; live birth rate: 26.32% vs. 43.90%, *p* < 0.01). First, the overall live birth rate of the PGT group was significantly higher than that of the ICSI group. Blastocyst stage embryos were transferred in the PGT group; when these embryos underwent PGT diagnosis, they showed normal chromosomes. Further analysis of the implantation rate and live birth rate was performed. Results showed that the outcomes of the PGT and ICSI groups based on whether calcium ionophore (A23187) activation was performed were inconsistent. Activation did not significantly affect the clinical pregnancy and live birth outcomes in the PGT group, whereas the implantation rate and live birth rate of the A-ICSI group were significantly lower than those in the ICSI group. One possible reason may be that patients from the A-ICSI group had some genetic defects. Cleavage stage embryos were used for ICSI transfer and were not screened for continued culture blastocysts. The presence of such defects may have resulted in an increase in chromosomal defects because PGT testing was not performed on ICSI embryos. Therefore, we were unable to confirm whether chromosome aneuploidy occurred in the transferred embryos or blastocysts. These results suggests that continuing blastocyst culture of cleavage stage embryos followed by PGT testing in patients who undergo calcium ionophore (A23187) activation may increase the implantation rate and live birth rate of transferred embryos.

Analysis of the gestational age and birth weight of infants between the PGT and A-PGT groups and between the ICSI and A-ICSI groups showed that calcium ionophore (A23187) activation did not affect the gestational age and birth weight of infants born in the PGT and ICSI groups. A follow-up study was performed on 237 cycles, 74 pregnancies, and 47 live births for patients who underwent calcium ionophore (A23187) activation. The results showed that the incidence of congenital malformations in the live births was within the normal range, suggesting that calcium ionophore (A23187) activation did not increase the incidence of congenital malformations (30). However, some adverse genetic effects may only appear in later stages of a child’s growth (31). Therefore, even though calcium ionophore (A23187) activation is a salvage measure for fertilization defects, its use in AOA does not have a long precedence and studies on its long-term effects on conceived children are lacking. Therefore, more follow-up studies are required. As the concentration and treatment duration of calcium ionophore (A23187) used by various fertility centers are different, multicenter studies and meta-analysis may be challenging. Additionally, there is no consensus regarding the selection of patients with fertilization failure for calcium ionophore (A23187) treatment. Therefore, guidelines regarding the selection of patients for calcium ionophore (A23187) activation must be developed in the future, and scientific research and follow-up studies should be carried out to obtain more detailed data to guide subsequent research and clinical work.

## 5 Conclusions

In the present study, we analyzed the chromosomal status of blastocysts fertilized through calcium ionophore (A23187) activation to reduce the chances of fertilization failure after ICSI and the offspring born after embryo transfer. Calcium ionophore (A23187) activation did not affect the proportion of early embryonic development and blastocyst aneuploidy; furthermore, it did not affect the gestational age and weight at birth. These findings provide a scientific basis for the clinical use of calcium ionophore (A23187) activation for the treatment of patients with ICSI fertilization failure.

## Data availability statement

The original contributions presented in the study are included in the article/**Supplementary Material**. Further inquiries can be directed to the corresponding authors.

## Ethics statement

The studies involving human participants were reviewed and approved by The Institutional Review Board of the First Affiliated Hospital of Zhengzhou University. The patients/participants provided their written informed consent to participate in this study.

## Author contributions

JZ: data collecting, data analysis, and drafting the article. GD-Y: concept and design the study, collecting data, and data analysis. TZ and JY-H: data collecting and data analysis. GY, JH-H, QH, HF and YB: collecting data. YS: conception and design the study. All authors participated in the revision of the manuscript. All authors contributed to the article and approved the submitted version.

## References

[1] Rosenwaks Z Pereira N. The pioneering of intracytoplasmic sperm injection: historical perspectives. Reproduction (2017) 154(6):F71–7. doi: 10.1530/REP-17-0308 29046342

[2] Yeste M Jones C Amdani SN Patel S Coward K. Oocyte activation deficiency: A role for an oocyte contribution? Hum Reprod Update (2016) 22(1):23–47. doi: 10.1093/humupd/dmv040 26346057

[3] Sanders JR Swann K. Molecular triggers of egg activation at fertilization in mammals. Reproduction (2016) 152(2):R41–50. doi: 10.1530/REP-16-0123 27165049

[4] Mu J Zhang Z Wu L Fu J Chen B Yan Z. The identification of novel mutations in PLCZ1 responsible for human fertilization failure and a therapeutic intervention by artificial oocyte activation. Mol Hum Reprod (2020) 26(2):80–7. doi: 10.1093/molehr/gaaa003 31953539

[5] Kashir J Nomikos M Lai FA. Phospholipase C zeta and calcium oscillations at fertilisation: The evidence, applications, and further questions. Adv Biol Regul (2018) 67:148–62. doi: 10.1016/j.jbior.2017.10.012 29108881

[6] Ferrer-Buitrago M Dhaenens L Lu Y Bonte D Vanden Meerschaut F De Sutter P. Human oocyte calcium analysis predicts the response to assisted oocyte activation in patients experiencing fertilization failure after ICSI. Hum Reprod (2018) 33(3):416–25. doi: 10.1093/humrep/dex376 29329390

[7] Zhao X Cheng D Yu H Li B. Progress of assisted oocyte activation. Chin J Hum Sexuality (2021) 30(4):102–5. doi: 10.3969/j.issn.1672-1993

[8] Egashira A Murakami M Haigo K Horiuchi T and Kuramoto T. A successful pregnancy and live birth after intracytoplasmic sperm injection with globozoospermic sperm and electrical oocyte activation. Fertil Steril (2009) 92(6):2037.e5–9. doi: 10.1016/j.fertnstert.2009.08.013 19800059

[9] Nikiforaki D Vanden Meerschaut F de Roo C Lu Y Ferrer-Buitrago M de Sutter P. Effect of two assisted oocyte activation protocols used to overcome fertilization failure on the activation potential and calcium releasing pattern. Fertil Steril (2016) 105(3):798–806.e2. doi: 10.1016/j.fertnstert.2015.11.007 26632207

[10] Hoshi K Yanagida K Yazawa H Katayose H and Sato A. Intracytoplasmic sperm injection using immobilized or motile human spermatozoon. Fertility Sterility (1995) 63(6):1241–5. doi: 10.1016/0020-7292(96)88083-0 7750594

[11] Xu Z Yao G Niu W Fan H Ma X Shi S. Calcium Ionophore (A23187) Rescues the activation of unfertilized oocytes after intracytoplasmic sperm injection and chromosome analysis of blastocyst after activation. Front Endocrinol (Lausanne) (2021) 12:692082. doi: 10.3389/fendo.2021.692082 34335469 PMC8320372

[12] Murugesu S Saso S Jones BP Bracewell-Milnes T Athanasiou T Mania A. Does the use of calcium ionophore during artificial oocyte activation demonstrate an effect on pregnancy rate? A meta-analysis. Fertil Steril (2017) 108(3):468–82.e3. doi: 10.1016/j.fertnstert.2017.06.029 28865547

[13] Miller N Biron-Shental T Sukenik-Halevy R Klement AH Sharony R and Berkovitz A. Oocyte activation by calcium ionophore and congenital birth defects: a retrospective cohort study. Fertil Steril (2016) 106(3):590–6.e2. doi: 10.1016/j.fertnstert.2016.04.025 27143515

[14] Capalbo A Ottolini CS Griffin DK Ubaldi FM Handyside AH Rienzi,L. Artificial oocyte activation with calcium ionophore does not cause a widespread increase in chromosome segregation errors in the second meiotic division of the oocyte. Fertil Steril (2016) 105(3):807–14.e2. doi: 10.1016/j.fertnstert.2015.11.017 26658129

[15] Dikensoy E Balat O Pence S Akcali C and Cicek H. The risk of hepatotoxicity during long-term and low-dose flutamide treatment in hirsutism. Arch Gynecol Obstet (2009) 279(3):321–7. doi: 10.1007/s00404-008-0719-z 18607612

[16] Wang N Ni JX and Zhuan L. Effects of Intracytoplasmic Sperm Microinjection on Offspring Epigenetics. Med Recapitulate (2020) 26(8):1505–9. doi: 10.3969/j.issn.1006-2084.2020.08.010

[17] Brinsden. Textbook of *In Vitro* Fertilization and Assisted Reproduction. Br Med J (Clin Res Ed) (1999) 2(2807):667–8. doi: 10.1016/S0015-0282(00)00474-X

[18] Gardner DK Lane M Stevens J Schlenker T Schoolcraft WB. Blastocyst Score Affects Implantation and Pregnancy Outcome: Towards a Single Blastocyst Transfer. Fertil Steril (2000) 73:1155–8. doi: 10.1016/S0015-0282(00)00518-5 10856474

[19] Niu W Wang L Xu J Li Y Shi H Li G. Improved clinical outcomes of preimplantation genetic testing for aneuploidy using MALBAC-NGS compared with MDA-SNP array. BMC Pregnancy Childbirth (2020) 20(1):388. doi: 10.1186/s12884-020-03082-9 32620095 PMC7333433

[20] Johnson LN Sasson IE Sammel MD Dokras A. Does intracytoplasmic sperm injection improve the fertilization rate and decrease the total fertilization failure rate in couples with well-defined unexplained infertility? A systematic review and meta-analysis. Fertil Steril (2013) 100(3):704–11. doi: 10.1016/j.fertnstert.2013.04.038 23773312

[21] Yanagida K Katayose H Hirata S Yazawa H Hayashi S Sato A. Influence of sperm immobilization on onset of Ca(2+) oscillations after ICSI. Hum Reprod (2001) 16(1):148–52. doi: 10.1093/humrep/16.1.148 11139554

[22] Kashir J Ganesh D Jones C Coward K. Oocyte activation deficiency and assisted oocyte activation: mechanisms, obstacles and prospects for clinical application. Hum Reprod Open (2022) 2022(2):hoac003. doi: 10.1093/hropen/hoac003 35261925 PMC8894871

[23] Ebner T Köster M Shebl O Moser M van der Ven H Tews G. Application of a ready-to-use calcium ionophore increases rates of fertilization and pregnancy in severe male factor infertility. Fertil Steril (2012) 98(6):1432–7. doi: 10.1016/j.fertnstert.2012.07.1134 22921909

[24] Sunderam S Boulet SL Kawwass JF Kissin DM. Comparing fertilization rates from intracytoplasmic sperm injection to conventional *in vitro* fertilization among women of advanced age with non-male factor infertility: A meta-analysis. Fertil Steril (2020) 113(2):354–63.e1. doi: 10.1016/j.fertnstert.2019.09.035 32106989 PMC10983030

[25] Aydinuraz B Dirican EK Olgan S Aksunger O Erturk OK. Artificial oocyte activation after intracytoplasmic morphologically selected sperm injection: A prospective randomized sibling oocyte study. Hum Fertil (Camb) (2016) 19(4):282–8. doi: 10.1080/14647273.2016.1240374 27734719

[26] Miao YL Stein P Jefferson WN Padilla-Banks E Williams CJ. Calcium influx-mediated signaling is required for complete mouse egg activation. Proc Natl Acad Sci U.S.A. (2012) 109(11):4169–74. doi: 10.1073/pnas.1112333109 PMC3306664 22371584

[27] Ebner T Montag M Oocyte Activation Study Group Montag M van der Ven K van der Ven H. Live birth after artificial oocyte activation using a ready-to-use ionophore: A prospective multicentre study. Reprod BioMed Online (2015) 30(4):359–65. doi: 10.1016/j.rbmo.2014.11.012 25596904

[28] Ebner T Sommergruber M Moser M Shebl O Schreier-Lechner E Tews AG. Basal level of anti-mullerian hormone is associated with oocyte quality in stimulated cycles. Hum Reprod (2006) 21(8):2022–6. doi: 10.1093/humrep/del127 16679324

[29] Lehmann P Vélez MP Saumet J Lapensée L Jamal W Bissonnette F. Anti-Müllerian hormone (AMH): A reliable biomarker of oocyte quality in IVF. J Assist Reprod Genet (2014) 31(4):493–8. doi: 10.1007/s10815-014-0193-4 PMC3969465 24573377

[30] Mateizel I Verheyen G Van de Velde H Tournaye H Belva F. Obstetric and neonatal outcome following ICSI with assisted oocyte activation by calcium ionophore treatment. J Assist Reprod Genet (2018) 35(6):1005–10. doi: 10.1007/s10815-018-1124-6 PMC6030021 29392515

[31] Santella L Dale B. Assisted yes, but where do we draw the line? Reprod BioMed Online (2015) 31(4):476–8. doi: 10.1016/j.rbmo.2015.06.013 26277587

