# Supplementary material for: Effect of calcium ionophore (A23187) on embryo development and its safety in PGT cycles
Source: Front Endocrinol (Lausanne). 2023 Jan 4;13:979248. doi: 10.3389/fendo.2022.979248 (PMC9846205; doi:10.3389/fendo.2022.979248)
Supplement: Supplementary file 1 [file Table_1.docx]

**Supplementary Table 1. The comparison of general clinical characteristics of patients between PGT and A-PGT group**

| **Groups** | **PGT** | **A-PGT** | **P value** |
| --- | --- | --- | --- |
| Age (year) | 30.31±2.62 | 28.75±4.35 | 0.099 |
| BMI (kg/m^2^) | 22.52±1.62 | 23.02±3.83 | 0.535 |
| AMH（ng/ml） | 4±2.47 | 5.2±3.54 | 0.121 |
| Basal FSH (mIU/ml) | 6.3±1.92 | 6.89±1.33 | 0.15 |
| Basal LH (mIU/ml) | 4.05±3.15 | 5.19±2.83 | 0.116 |
| Basal E_2_ (pg/ml) | 37.46±15.09 | 40.59±18.52 | 0.439 |
| Basal P (ng/ml) | 0.72±1.16 | 0.42±0.32 | 0.219 |
| Basal PRL (ng/ml) | 14.94±5.73 | 16.21±4.91 | 0.317 |
| Basal T (ng/ml) | 0.3±0.16 | 0.35±0.23 | 0.207 |
| FT3 (pmol/ml) | 5.15±0.64 | 5.27±0.76 | 0.413 |
| FT4 (pmol/ml) | 11.78±2.16 | 12.43±2.74 | 0.18 |
| TSH (uIU/ml) | 2.28±1.09 | 3±1.81 | 0.068 |

Data are expressed as the means ± standard deviation.
