# Supplementary material for: Effect of calcium ionophore (A23187) on embryo development and its safety in PGT cycles
Source: Front Endocrinol (Lausanne). 2023 Jan 4;13:979248. doi: 10.3389/fendo.2022.979248 (PMC9846205; doi:10.3389/fendo.2022.979248)
Supplement: Supplementary file 2 [file Table_2.docx]

**Supplementary Table 2. The comparison of general clinical characteristics of patients between group of A-ICSI and A-PGT**

| **Groups** | **A-ICSI** | **A-PGT** | **p value** |
| --- | --- | --- | --- |
| No. | 76 | 24 | - |
| Age (year) | 29.26±3.78 | 28.75±4.35 | 0.577 |
| BMI (kg/m^2^) | 23.52±3.06 | 23.02±3.83 | 0.508 |
| AMH（ng/ml） | 3.62±2.63 | 5.2±3.54 | 0.024^*^ |
| Basal FSH (mIU/ml) | 6.65±2.01 | 6.89±1.33 | 0.587 |
| Basal LH (mIU/ml) | 4.9±3.13 | 5.19±2.83 | 0.701 |
| Basal E_2_ (pg/ml) | 34.41±14.16 | 40.59±18.52 | 0.129 |
| Basal P (ng/ml) | 0.41±0.32 | 0.42±0.32 | 0.884 |
| Basal PRL (ng/ml) | 18.31±7.39 | 16.21±4.91 | 0.128 |
| Basal T (ng/ml) | 0.29±0.16 | 0.35±0.23 | 0.157 |
| FT3 (pmol/ml) | 5.21±0.66 | 5.27±0.76 | 0.71 |
| FT4 (pmol/ml) | 11.38±1.55 | 12.43±2.74 | 0.082 |
| TSH (uIU/ml) | 2.47±1.13 | 3±1.81 | 0.185 |

Data are expressed as the means ± standard deviation. * *p* < 0.05.
