# Supplementary material for: Effect of calcium ionophore (A23187) on embryo development and its safety in PGT cycles
Source: Front Endocrinol (Lausanne). 2023 Jan 4;13:979248. doi: 10.3389/fendo.2022.979248 (PMC9846205; doi:10.3389/fendo.2022.979248)
Supplement: Supplementary file 3 [file Table_3.docx]

**Supplementary Table 3. The comparison of general clinical characteristics of patients between groups of ICSI and A-ICSI**

| **Groups** | **ICSI** | **A-ICSI** | **p value** |
| --- | --- | --- | --- |
| No. | 234 | 76 | - |
| Age (year) | 30.13±2.61 | 29.26±3.78 | 0.065 |
| BMI (kg/m^2^) | 22.78±1.68 | 23.52±3.06 | 0.045^*^ |
| AMH（ng/ml） | 4.07±2.75 | 3.62±2.63 | 0.227 |
| Basal FSH (mIU/ml) | 6.16±1.81 | 6.65±2.01 | 0.048^*^ |
| Basal LH (mIU/ml) | 5.35±3.52 | 4.9±3.13 | 0.32 |
| Basal E_2_ (pg/ml) | 38.66±16.84 | 34.41±14.16 | 0.061 |
| Basal P (ng/ml) | 0.39±1.25 | 0.41±0.32 | 0.907 |
| Basal PRL (ng/ml) | 17.2±7.2 | 18.31±7.39 | 0.269 |
| Basal T (ng/ml) | 0.27±0.14 | 0.29±0.16 | 0.459 |
| FT3 (pmol/ml) | 5.25±0.65 | 5.21±0.66 | 0.643 |
| FT4 (pmol/ml) | 11.46±1.67 | 11.38±1.55 | 0.71 |
| TSH (uIU/ml) | 2.46±1.2 | 2.47±1.13 | 0.952 |

Data are expressed as the means ± standard deviation. * *p* < 0.05.
