# Supplementary material for: Effect of calcium ionophore (A23187) on embryo development and its safety in PGT cycles
Source: Front Endocrinol (Lausanne). 2023 Jan 4;13:979248. doi: 10.3389/fendo.2022.979248 (PMC9846205; doi:10.3389/fendo.2022.979248)
Supplement: Supplementary file 4 [file Table_4.docx]

**Supplementary Table 4. Comparison of offspring birth between group of ICSI and A-ICSI**

| **Groups** | | **ICSI** | **A-ICSI** | **p value** |
| --- | --- | --- | --- | --- |
| Singleton | NO. | 66 | 10 | - |
|  | Birth (wk) | 39.04±1.63 | 38.44±2.57 | 0.322 |
|  | Weight (g) | 3443.29±495.49 | 3142.00±714.51 | 0.096 |
| Twin | NO. | 60 | 10 | - |
|  | Birth (wk) | 36.88±1.73 | 37.00±1.77 | 0.888 |
|  | Weight (g) | 2561.67±458.86 | 2474.00±635.68 | 0.528 |

*p* < 0.05 indicates statistically significant differences.
